# Infective Endocarditis During Pregnancy: Challenges and Future Directions

**DOI:** 10.3390/jcm14124262

**Published:** 2025-06-16

**Authors:** Eleni Polyzou, Evangelia Ntalaki, Dimitrios Efthymiou, Despoina Papageorgiou, Maria Gavatha, Emmanouil Angelos Rigopoulos, Katerina Skintzi, Stamatia Tsoupra, Konstantinos Manios, Nikolaos G. Baikoussis, Karolina Akinosoglou

**Affiliations:** 1 Department of Medicine, University of Patras, 26504 Patras, Greece; polyzou.el@gmail.com (E.P.); dspn.pap96@gmail.com (D.P.); gavatha.maria@yahoo.com (M.G.); agrigopoulos@gmail.com (E.A.R.); stamtsoupra@gmail.com (S.T.); 2 Department of Internal Medicine, University General Hospital of Patras, 26504 Rio, Greece; efthdim2@gmail.com; 3 Department of Obstetrics and Gynecology, University General Hospital of Patras, 26504 Rio, Greece; evangelia.ntalaki@gmail.com; 4 Department of Infection Control, University General Hospital of Patras, 26504 Rio, Greece; katrinski80@gmail.com; 5 Department of Cardiac Surgery, Ippokrateio General Hospital of Athens, 11527 Athina, Greece; manios88@hotmail.com (K.M.); nikolaos.baikoussis@gmail.com (N.G.B.); 6 Division of Infectious Diseases, University General Hospital of Patras, 26504 Rio, Greece

**Keywords:** endocarditis, pregnancy, bacteremia, native valve, *Staphylococcus*

## Abstract

Infective endocarditis (IE) during pregnancy, while uncommon, is associated with substantial maternal and fetal morbidity and mortality due to the complex physiological adaptations of pregnancy. Hemodynamic alterations, including increased cardiac output and changes in vascular resistance, combined with immunological modulation, predispose pregnant individuals to increased risk of infection and associated complications. Predominant pathogens implicated in pregnancy-associated IE are *Staphylococcus aureus*, *Streptococcus viridans*, and *Enterococcus faecalis*, with *S. aureus* infections frequently leading to poorer clinical outcomes. Diagnosis remains challenging due to commonly atypical presentation and relies on microbiological identification via blood cultures in conjunction with imaging modalities such as transthoracic echocardiography. IE in pregnancy is associated with increased maternal mortality rates (5–17%) and adverse fetal outcomes, including preterm birth, intrauterine growth restriction (IUGR), and fetal loss. Management necessitates careful selection of antimicrobial therapy to ensure efficacy while minimizing fetal toxicity, especially in settings of increased antimicrobial resistance. Anticoagulation and surgical interventions must be judiciously considered, with surgical timing individualized based on the severity of heart failure and coordinated multidisciplinary care. In conclusion, IE during pregnancy constitutes a significant clinical challenge, underscoring the need for enhanced diagnostic strategies, optimized therapeutic protocols, and the development of pregnancy-specific management guidelines to improve maternal and fetal outcomes.

## 1. Introduction

Infective endocarditis (IE) is a rare condition characterized by a bacterial, viral, or fungal infection of the endocardial surfaces of the heart—most commonly affecting one or more cardiac valves, but also, to a lesser extent, the mural endocardium or a septal defect [1]. IE can originate either in the community or following exposure in a healthcare setting. Community-associated IE occurs when the infection develops without recent interaction with a healthcare facility and is diagnosed within 48 h of hospital admission. In contrast, healthcare-associated IE arises following recent exposure to a medical setting, with symptoms emerging at least 48 h after hospitalization [2]. Despite being rare, IE remains associated with high morbidity and mortality. It is currently the third or fourth most life-threatening infectious syndrome, following sepsis, pneumonia, and intra-abdominal abscess [3]. IE is associated with serious complications such as embolic events and heart failure, both of which significantly impact patient outcomes and prognosis. In fact, in studies that addressed the impact of those complications on prognosis in IE, embolic events and IE-associated heart failure were found to be independent predictors of major adverse events, including death, hospitalizations, and relapse. Notably, heart failure showed a particularly strong association with all-cause mortality [4].

IE remains a severe and potentially life-threatening condition, particularly in high-risk populations, including pregnant individuals. Although considered rare, with an incidence of approximately 0.006% [5], IE in pregnancy has been reported with increasing frequency, corresponding to the rise in cases among both pregnant and non-pregnant individuals due to the increasing use of intravenous opioids [6]. The cardiovascular changes that occur during pregnancy, along with physiologic and immune adaptations, may increase susceptibility to complications and the risk of hemodynamic instability, particularly in those with valvular disease, making IE a serious concern during both pregnancy and the postpartum period [7]. Additionally, the need to consider both maternal and fetal health further complicates management [7]. While precise mortality rates remain unclear, estimates range from 5–33% [6]. However, advancements in early diagnosis and treatment, including medical and surgical interventions, have contributed to improved outcomes [8]. Due to the absence of randomized controlled trials involving pregnant individuals, there is a significant gap in the comprehensive understanding of IE during the antepartum and postpartum periods. This lack of data limits knowledge regarding the management of feto-maternal complications, largely because of the condition’s low reported incidence [9].

This literature review aims to summarize available data on IE during pregnancy, identifying risk factors that increase susceptibility, highlighting differences in diagnosis, exploring the limited treatment options, examining the unique aspects of surgical interventions, and discussing future perspectives for improving management in this high-risk population.

## 2. Methodology

As part of a scoping review, a comprehensive literature search was conducted using PubMed to identify relevant clinical studies, case series, and systematic reviews evaluating the presentation, management, and outcomes of IE during pregnancy. The search strategy was developed using a combination of terms related to IE and its etiologies—including microbial agents, diagnostic modalities, and risk factors—and terms associated with pregnancy and related maternal–fetal outcomes. The search string employed included “infective endocarditis,” “*Staphylococcus aureus*,” “bacteremia,” “prosthetic valve,” “transthoracic echocardiography,” “transesophageal echocardiography,” “congenital heart disease,” “rheumatic heart disease,” “streptococci,” “staphylococci,” “enterococci,” “*Haemophilus* spp.,” “*Candida* spp.,” “anticoagulant therapy,” “valve surgery,” “antibiotic prophylaxis” AND “pregnancy,” “fetus,” “maternal complications,” “postpartum period,” “fetal complications,” “trimester,” “intrauterine growth restriction,” “neonatal sepsis,” “preterm labor,” “miscarriage,” and “maternal mortality.” Exclusion criteria included non-English publications, studies involving non-human subjects, articles not published in peer-reviewed journals, and non-systematic narrative reviews.

In total, 11 independent reviewers collaboratively conducted the literature screening and article selection process. Each reviewer assessed the titles and abstracts of studies retrieved from the initial search to identify those that met the inclusion criteria. Full texts of potentially relevant articles were then examined in detail. Eligible studies included clinical studies, case series, and systematic reviews that focused on infective endocarditis during pregnancy, with particular attention to microbial etiology, diagnostic methods, maternal and fetal outcomes, and therapeutic approaches. Studies reporting on general cases of endocarditis were also considered if they included a pregnant population or stratified results by pregnancy status. Additional literature was identified through manual screening of references and related citations on PubMed.

**Figure 1:**
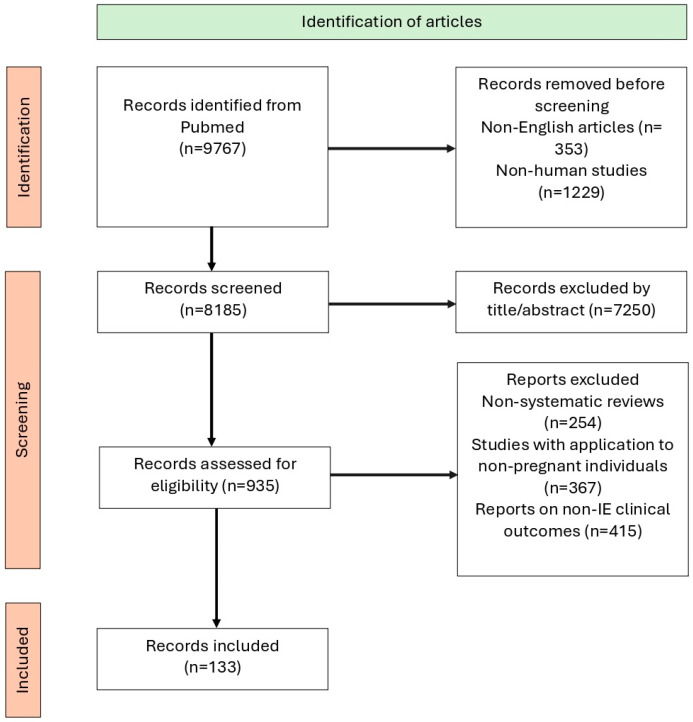
The selection process using a PRISMA flowchart.

The study selection process was documented using a PRISMA flowchart (Figure 1), illustrating each stage of identification, screening, and inclusion.

## 3. Epidemiology and Risk Factors

The incidence of IE, although rare, has significantly increased, reaching 1,090,530 cases in 2019 compared to 478,000 cases in 1990 [10], now corresponding to 3 to 9 cases per 100,000 individuals annually [11]. This remarkable global rise in incidence is attributed to epidemiological changes and technological advancements driven by global industrialization [9]. Leading factors contributing to this increase include the opioid epidemic, particularly in high-income countries, as well as the growing use of prosthetic valves and other cardiac devices [12].

As previously mentioned, the incidence of IE during pregnancy is extremely low (0.006%) and considerably lower compared to the general population. The predisposing factors for IE during pregnancy are not significantly different from those in the general population, with three distinct groups being prominent: congenital heart disease (12%), intravenous drug use (14–23%), and rheumatic heart disease (12%) [8,13]. Congenital heart diseases, such as ventricular wall defects, tetralogy of Fallot, and bicuspid aortic valves, increase the likelihood of developing IE in pregnancy by 50%, making the heart valves more susceptible to infection through pathological blood flow and endothelial damage [14]. Similarly, the presence of a prosthetic valve poses a significant risk for IE, as the prosthetic material may allow bacterial attachment during bacteremia. In pregnant women with congenital heart disease, the incidence of IE increases to 1 per 1000 pregnancies, while in those with prosthetic heart valves, the incidence reaches 3 to 12 cases per 1000 pregnancies [9].

Although rheumatic heart disease has declined in high-income regions, it remains a significant threat in middle- and low-income countries. Pregnant women with rheumatic valvular diseases, such as mitral valve insufficiency or stenosis, are at high risk of developing IE due to turbulent blood flow through the damaged valves [13]. In low socioeconomic regions, delayed or inadequate access to healthcare facilities, coupled with the lack of advanced cardiac surgical interventions, results in a higher incidence of complications and mortality associated with IE [15]. Last, women with a history of IE also exhibit an increased risk of relapse, complications, and mortality during pregnancy [16].

Historically, congenital and rheumatic heart diseases were the primary etiologies of IE in young individuals. However, with technological advancements in the past two decades, there seems to be a shift in the prevalence of risk factors. Chronic comorbidities, such as hypertension, diabetes, and chronic kidney disease, along with increasing age, are gradually replacing classical risk factors like immunosuppression, intravenous access, prosthetic valves, and hemodialysis as the leading contributors to the incidence of IE [17]. Moreover, in pregnancy, invasive procedures that carry a risk of bacteremia, particularly when aseptic techniques are compromised, such as urinary catheterization, epidural anesthesia, and obstetric interventions, including Cesarean sections and amniocentesis, significantly contribute to the risk of IE. Additionally, membrane rupture, prolonged labor, and manual removal of the placenta further exacerbate this risk [18]. The use of contaminated equipment (such as needles) and the disruption of aseptic techniques, often associated with intravenous drug use (IVDU), remains the primary extracardiac risk factor for IE during the perinatal period, with an estimated incidence of 23%, according to current literature [19]. In this setting, the tricuspid valve is most commonly affected, with *S. aureus* being the predominant microorganism [19].

## 4. Pathophysiology of IE in Pregnancy

IE is a condition characterized by inflammation of the heart’s inner lining due to an infection. This infection develops as a result of complex interactions between pathogenic microorganisms and the body’s immune response. IE pathogenic microorganisms like *S. aureus* and *S. viridans* preferentially colonize biofilm-coated regions on the cardiac valves. Within these biofilms, they interact through complex biological systems, cooperating to enhance survival by resisting phagocytosis from immune cells and increasing tolerance to antibiotic treatments [14,20].

Pregnancy leads to hemodynamic changes, including a 30–40% increase in cardiac output and a substantial expansion in plasma volume [20,21]. As a result, the normal blood flow of heart is altered, leading to turbulence across the heart valves. Similarly, individuals with pre-existing valvular defects experience abnormal turbulent blood flow, which can further disrupt normal circulation. This turbulence contributes to endothelial injury, creating a favorable environment for complications such as infection or clot formation [22]. In this context, pregnancy is typically characterized by high levels of clotting factors, such as Von Willebrand factor and fibrinogen, and low levels of antithrombin III and protein S, contributing to a hypercoagulable state [23,24].

As pregnancy progresses into the third trimester, for most individuals, the growing uterus mechanically compresses the inferior vena cava [16]. This consequently impairs venous blood return, leading to increased vascular congestion [22,25]. The impaired blood flow leads to increased endothelial stress, which promotes bacterial colonization [26]. The hemodynamic stress exerted by the growing uterus in late pregnancy is particularly pronounced in individuals with pre-existing structural heart abnormalities, significantly raising the risk of IE [8,26].

Maternal immune system undergoes significant changes immediately after labor as it returns to the baseline status prior to pregnancy. This phase of post-pregnancy is marked by an increase in pro-inflammatory responses, the reestablishment of the endothelial dysfunction caused by pregnancy, and the normalization of coagulation function. These changes can activate autoimmune diseases in susceptible individuals or aid the resolution of pregnancy-associated infections [27,28]. The heightened immune reconstitution during this period raises concerns about maternal health following delivery [26].

Additionally, pregnancy induces a shift in immune function to achieve fetal tolerance, which involves a decrease of the protective Th1 immune response and a shift toward an anti-inflammatory Th2 type with an enhanced regulatory T cell (Treg) activity [29]. While these changes help prevent fetal rejection, they also decrease maternal immunity, impairing the ability to clear bacteria, thus increasing susceptibility to infections such as IE [30].

Furthermore, placental-derived factors, such as soluble feto-placental antigens and extracellular vehicles (EVs), not only help maintain immune tolerance but also sustain systemic hypercoagulation and endothelial damage. Soluble antigens like HLA-G inhibit the activity of natural killer (NK) cells and dendritic cells, while placental EVs carry immunosuppressive microRNAs (miRNAs) that downregulate genes involved in pathogen recognition and immune activation [18,22].

Another factor is the alteration of the maternal microbiome during pregnancy, which also influences the immune responses and the body’s ability to fight back bacterial infections [21,29]. An imbalance in the healthy microorganisms of the oral cavity or gastrointestinal tract can allow various bacteria to enter the bloodstream, increasing the risk of developing IE. This is especially relevant for *S. viridans* IE, which primarily originates from the oral cavity [29].

Pregnancy hormonal changes further modify the immune function. Εlevated levels of progesterone in pregnancy downregulate the production of Th1-type cytokines and promote anti-inflammatory cytokine production, such as IL-10 [31,32], which reduces the body’s ability to fight bacterial infections. On the other hand, estrogen modulates vascular tone and endothelial integrity, creating a microenvironment that promotes bacterial adherence and biofilm formation [14].

The pathophysiology of vegetation formation in IE can vary depending on the infecting organism. Typically, endocardial damage exposes adhesion molecules, followed by platelet and fibrin attachment, leading to vegetation formation [33,34]. Initially, a sterile platelet–fibrin nidus becomes contaminated by blood pathogens, often from distant infections or temporary bacteremia from mucosal or cutaneous sources [35,36]. Pregnancy-related injury of endothelial cells can occur due to oxidative stress and limited nitric oxide [36,37]. Microbial growth activates the extrinsic clotting pathway, and monocytes adhere to releasing cytokines, while stimulated endothelial cells deposit more fibronectin. In these vegetations, bacteria proliferate within cells and fibronectin matrix, making it difficult for host immune responses to manage or eliminate the infection [36]. *S. aureus*, for example, may bind to endothelial cells via extracellular matrix binding proteins, internalize, and then avoid the host immune response. Once inside, it can activate endothelial cells and destroy tissue through the secretion of exoproteins [36].

**Figure 2:**
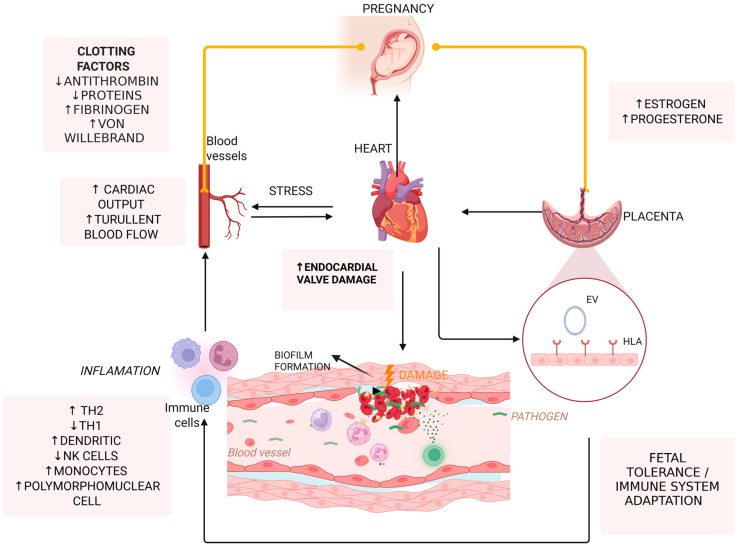
Pathophysiology of IE during pregnancy. ↑: increase; ↓: decrease.

Figure 2 summarizes main mechanisms underlying pathophysiology of IE in pregnancy.

## 5. Microbiology of Pregnancy-Associated IE

The causative pathogens of IE do not differ between pregnant and non-pregnant individuals [9]. Streptococci (approximately 40–43%), staphylococci (approximately 20–29%), and enterococci (approximately 12%) represent the majority of pathogens causing ΙΕ, with *S. aureus* being an increasingly isolated pathogen, associated with worse prognosis [8,9,14,38]. The *S. viridans* group also represents a high prevalent pathogen as they are implicated in approximately 18.9% of IE cases in pregnancy [38]. They are part of the normal oral flora, and they can cause IE typically following dental procedures, especially in individuals with underlying cardiac conditions such as congenital heart disease, valve replacement, or history of previous IE [39,40]. Noteworthy is that streptococcal endocarditis affects mainly the left side of the heart, whereas staphylococcal endocarditis affects mainly the right side of the heart [14].

**Figure 3:**
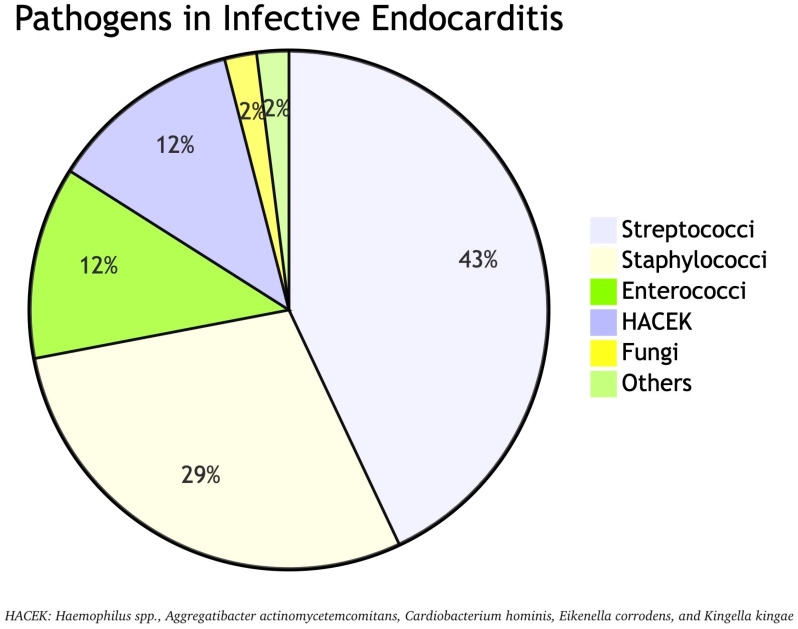
Pathogens in infective endocarditis.

Moreover, enterococci are the third most common group, following staphylococci and streptococci. Among them, *E. faecalis* is the most frequently implicated pathogen in the development of IE. Of note, enterococcal IE is more common in elderly and those with prosthetic valve implants [41]. Another group of microorganisms that rarely cause IE, but are among the most common Gram-negative pathogens involved, is the HACEK (*Haemophilus* spp., *Aggregatibacter actinomycetemcomitans*, *Cardiobacterium hominis*, *Eikenella corrodens*, and *Kingella kingae*) organisms reaching a percentage of 12% [12]. They are part of the normal flora of the oral and upper respiratory tract and, when properly treated, have excellent outcomes [42]. Although the etiology of IE during pregnancy is mostly of bacterial origin, fungal endocarditis should also be considered. The most common fungal pathogens involved are *Candida* spp., followed by *Aspergillus* and *Histoplasma* spp., accounting for approximately 1–2% of all IE cases [14,43]. Figure 3 illustrates the proportional distribution of each pathogen identified in cases of IE.

## 6. Screening and Risk Assessment for High-Risk Pregnant Patients—Is There a Role?

While rare (1 per 100,000 pregnancies) [44], IE is a problem due to the unique physiological changes that occur in pregnancy, which may alter the course and presentation of the disease. Early identification of high-risk pregnant patients is vital for improving maternal and fetal outcomes.

Risk stratification tools, which assess factors like structural heart disease, diabetes, and a history of recent surgeries, can guide clinicians in identifying high-risk individuals. There are several risk factors for IE during pregnancy, including congenital heart disease, valvular heart disease, IVDU, prosthetic heart valves, prior history of IE, and immunocompromise [14,17,45,46,47]. Although the 2023 European Society of Cardiology (ESC) Guidelines for the management of endocarditis does not recommend regular screening for pregnant women, diagnosis should be considered in pregnant women with unexplained tachycardia, fever, new or changing cardiac murmurs, and peripheral signs of septic emboli.

The gravity of the condition requires the inclusion of gynecologists, obstetricians, and neonatologists in any suspected cases. A diagnosis and treatment plan have to be formulated rapidly, as this is key to saving the lives of mothers and infants [48].

## 7. Clinical Presentation and Diagnostic Challenges

Diagnosis of IE in pregnancy is challenging due to the changing cardiovascular physiology, characterized by prolonged, moderate tachycardia, high preload, and low afterload, which can mimic the clinical picture of cardiovascular diseases [49]. Hemodynamic alternations and various changes in cardiac valves can lead to the presence of murmurs, which can be challenging to interpret during physical examination [9]. Clinical presentation of IE in pregnant patients can range from subacute/chronic infection, with low-grade fever and non-specific symptoms to acute rapidly progressive infection. The disease should be considered in any pregnant patient with unexplained fever, night sweats, systemic illness, embolic manifestation, or cardiac murmur. Classical signs of IE, including petechiae, Roth spots, Osler’s nodules, and Janeway lesions, may be observed in subacute cases. In some cases, the disease may present initially with its complications, including heart failure or embolic manifestations [48,49,50].

A recent study comparing the clinical presentation of IE among pregnant and non-pregnant individuals found that, overall, the presentation was similar between the two study arms [7]. However, there was an increased incidence of shortness of breath and a decreased incidence of stroke and altered mental status among pregnant women [7]. Also, pregnant women were less likely to present with leukocytosis [7]. The incidence of fever was similar in both study groups; however, in pregnant individuals, fever requires special attention as it is frequently associated with various conditions, including chorioamnionitis, pneumonia, viral infections, and pyelonephritis [14].

The American Heart Association (AHA) and the ESC do not recommend any special adjustments in the diagnostic approach of IE in pregnant patients [12,48]. Diagnosis primarily relies on the presence of major or minor parameters outlined in Duke’s criteria, which classify IE cases into definitive, possible, or rejected [9]. Positive blood cultures remain the cornerstone of diagnosis, with recent guidelines recommending at least three sets of blood cultures obtained at 30-min intervals prior to antibiotic therapy initiation [48].

Additionally, imaging assessment is essential for diagnosis, management, and prognosis of IE. AHA, ESC, and the American College of Obstetricians and Gynecologists (ACOG) recommend transthoracic echocardiography (TTE) and magnetic resonance imaging (MRI) without contrast, as first-line options for cardiac imaging in the setting of pregnancy [51]. Conversely, the role of transesophageal echocardiography (TEE) and computed tomography (CT) imaging is debated in pregnancy due to the heightened risk of emesis, aspiration, and radiation exposure considerations, as well as the risks associated with the use of anesthetic agents [51].

## 8. Maternal and Fetal Complications

IE represents a serious infection that can complicate pregnancy potentially leading to significant fetal and maternal complications. Pregnancy-associated IE carries a significant maternal mortality risk, estimated between 8.1% and 17.2%, which is comparable to, or even higher than, IE in the non-pregnant population [52]. The increased mortality is primarily due to cardiovascular complications, including valve destruction, severe heart failure, and septic embolization, leading to stroke or multiorgan failure [53]. A large-scale retrospective cohort study indicated that pregnancy-associated IE has comparable mortality rates to non-pregnancy-related cases (8.1% vs. 10.6%) [54]. However, the mortality rate significantly rises when IE complicates delivery, with reports suggesting up to 17.2% maternal mortality compared to <0.01% in normal deliveries [54]. The higher mortality in the peripartum period is likely due to hemodynamic instability, increased risk of sepsis, and thromboembolic complications [55].

When combined with the immunologic and hemodynamic changes during pregnancy and the weeks following delivery, the risks associated with IE are increased. As already discussed above, during pregnancy, the maternal immune profile shifts, with a reduced inflammatory status observed in the second trimester. Hemodynamically, a high-pressure environment is created, as cardiac output increases by up to 45% in the third trimester and heart rate steadily rises by 25% [14]. This elevated pressure can cause vegetation to dislodge, leading to systemic or pulmonary embolism [56]. Septic embolization is a significant concern, with embolic stroke occurring in up to 30% of cases, leading to significant neurological morbidity [57]. Pregnant patients with IE are also more likely to develop severe complications, such as septic shock, respiratory failure [58], prosthetic valve dysfunction, mycotic aneurysms, and perivalvular abscess formation, further increasing the complexity of management [12]. Possible complications on the fetus include preterm labor, intrauterine growth restriction (IUGR), neonatal sepsis, miscarriage, and fetal loss [5].

An area that should also be kept in mind when referring to IE during pregnancy is the cardiovascular health during and after pregnancy both for the fetus and the mother. Fetal toxicity is exacerbated by the existence of valvular vegetations or hemodynamic instability when the maternal cardiac reserve cannot meet pregnancy demands [59,60]. Decreased stroke volume and systolic dysfunction, even in the presence of preserved heart rate, are directly correlated with fetal growth restriction [61,62]. Fetal hemodynamic may be compromised when maternal cardiac output becomes ceilinged or diminished in the later part of gestation, a feature present in women with structural or acquired heart disease [63,64]. Subclinical or chronic inflammatory states contribute to these effects by amplifying endothelial dysfunction and altering vascular resistance in the uteroplacental unit, causing adverse neonatal outcomes like small-for-gestational-age infant and low Apgar score after labor [65,66]. Latent maternal cardiovascular dysfunction can be further destabilized by bidirectional fetal–maternal transmission, which includes metabolic demands and stress signals produced by the placenta. According to these results, the fetus is an active pathophysiologic agent that has the ability to adjust at any maternal cardiovascular modulation [59,67].

Admittedly, cardiovascular risk factors can mitigate adverse pregnancy outcomes with impact on subsequent cardiovascular health of mother and offspring. The evaluation of life’s essential “8” is crucial to assess in order to minimize risk in advance [68]. These eight factors are diet, physical activity, smoking status, body mass index, blood pressure, lipids, blood sugar, and sleep health. Added on, mental well-being, socioeconomic factors, and access to healthcare and resources also affect an individual’s health and can promote cardiovascular health. These sections mainly compose the possible areas of pre-existing maternal unfavorable cardiometabolic phenotype. The maternal environment can affect offspring’s cardiovascular health through a complex mechanism called “developmental programming” [68]. The three significant maternal conditions that can affect infant’s growth are pregnancy-related cardiovascular disorders (e.g., peripartum cardiomyopathy), maternal cardiovascular risk factors just previously mentioned, and extracardiac disorders (e.g., malnutrition and corticosteroid therapy). Later in life, this adverse developmental programming, along with lifestyle risk factors and genetic predisposition, can accelerate the development of cardiovascular risk factors and cardiovascular disease in childhood and adolescence [68].

Fetal inflammatory response syndrome (FIRS), which is another entity that can complicate infections during pregnancy and lead to severe fetal implications, is an area of major interest and concern. More specifically, FIRS represents the fetal inflammatory reaction to intrauterine infection, potentially leading to multiorgan impairment, neonatal mortality, and morbidity. It may occur after direct fetal exposition to inflammation in the amniotic fluid or through placental–fetal blood circulation, resulting in chorioamnionitis (CA), the inflammation of amnion and chorion. It can be caused by a microbial infective process that may follow ascending, transabdominal, or hematogenous pathways in case of bacteremia in IE [69]. The consequences of FIRS are preterm delivery mediated by pro-inflammatory cytokines or even fetal death due to hypoxic-ischemic encephalopathy. Moreover, postnatal complications are highly probable, too [69]. Infant neurological damage is the main postnatal complication due to neonatal encephalopathy, which is increased twelve-fold in term infants, leading to long-term disabilities such as cerebral palsy, cognitive impairment, epilepsy, blindness, deafness, and speech/language disorders, or even death in the neonatal period in up to 20% of cases, due to severe neurological disorders [69]. Finally, severe immunological cascades due to maternal infection can also mediate epigenetic changes in the fetal genome and affect the development of the fetus, leading to defects, with the most common ones being craniofacial and cardiac [70].

Based on recent guidelines of 2023 of ESC, IE is estimated to complicate ~1 in 100,000 pregnancies, with a maternal mortality of 18% mainly due to heart failure or embolization, while preterm birth is reported at 55.7% and fetal mortality at 29% [48]. However, due to IE rarity and the lack of major trials in pregnancy, little is known about the outcomes in population with case series and retrospective reviews being the main source of data (Table 1) [7,54].

A retrospective cohort study from the United States (1999–2014) showed that, out of 13,219,726 deliveries, 475 cases of IE were identified, corresponding to a rate of 3.6 per 100,000 deliveries [71]. Mortality in IE cases was 5.3% versus 0.003% in all other pregnant women. Key risk factors for death included acute renal failure, acute myocardial infarction, and sepsis [71]. A study from 2007 to 2018 on drug-injecting individuals with IE revealed poor outcomes for pregnant women. Of 11 pregnant women with IE, 27% died, 46% had elective terminations, and 18% miscarried. Of the 6 live births, the average gestational age was 35.3 weeks, with 83% delivered vaginally. Fetal mortality was 25% in pregnancies that were not electively terminated [72]. In another large national cohort study (2015–2018) involving 12,602 reproductive-aged women, 382 maternity-associated IE cases (3% of the total) were identified. Of these, 39.1% occurred antepartum, and 19% occurred postpartum. Mortality was similar between maternity-associated and non-maternity-associated IE, but the latter showed a higher rate of valve replacements in postpartum cases. Pregnancy complications associated with IE included maternal mortality (17.2%), preterm birth (55.7%), and a higher likelihood of C-sections. Preterm births were particularly common, with an average gestational age of 32.4 weeks [54].

In single-center reviews, right-sided endocarditis and tricuspid valve involvement were frequent, particularly in pregnant intravenous drug users. Maternal survival was generally good, but fetal outcomes were poor, with preterm births, low birth weight, and high fetal mortality. Studies also showed high rates of septic emboli, shock, and mechanical ventilation among IVDU women with IE, with maternal mortality as high as 22% [7,73,74].

Other studies reported similar findings: tricuspid valve involvement was common, and most cases were managed with antibiotics. While maternal mortality was low, complications included heart failure requiring surgery and neonatal complications like respiratory distress and low birth weight. *S. aureus*-related IE often led to septic emboli, severe sepsis, and respiratory failure but had relatively low maternal mortality (7%) due to multidisciplinary care [6,13,75]. Lastly, a multinational study of non-IVDU pregnant women with IE revealed a predominance of left-sided valve involvement (mitral and aortic), with heart failure being the most common complication. Despite maternal mortality was low, pregnancy-related complications were frequent, and emergency C-sections were frequent [38].

In conclusion, maternal mortality in pregnancy-associated IE ranges from 5–11%, and fetal mortality rates from 0–16%. Maternal mortality has decreased over time, likely due to a higher number of younger IVDU individuals with right-sided endocarditis, which tends to have a better prognosis. In the United States, fetal mortality due to maternal endocarditis is estimated at 14.6%, while maternal mortality ranges from 10–33% [8,73] (Supplemental Table S1).

## 9. Management Strategies

### 9.1. Antimicrobial Therapy

#### 9.1.1. General Principles

In the management of IE during pregnancy, the therapeutic approach largely mirrors that of non-pregnant patients. In line with ESC guidelines, bactericidal antibiotics is the preferred choice, with EUCAST MIC breakpoints being used to determine whether bacteria are susceptible, require increased exposure, or are resistant to antibiotics [48]. Nevertheless, concerns regarding potential fetal toxicity limit the range of therapeutic options, while careful consideration of the unique pharmacokinetic and pharmacodynamic alterations associated with pregnancy should be made [76].

In 1979, the United States Food and Drug Administration (FDA) classified drugs into five categories based on their safety for use during pregnancy. Category A drugs have been proven safe through controlled studies in pregnant women, showing no risk to the fetus. Category B drugs have either shown no harm in animal studies with no human studies available or have shown adverse effects in animals but not in human studies, making them generally considered safe. Category C and D drugs present uncertain or clear evidence of risk to the human fetus, respectively. Lastly, Category X drugs are strictly contraindicated in pregnancy, as studies have demonstrated definite fetal abnormalities, making their use unsafe for pregnant women [77]. The FDA’s Pregnancy and Lactation Labeling Rule (PLLR) replaces the outdated letter category system (A, B, C, D, X) with detailed narrative sections that provide more comprehensive and clinically relevant information. Instead of assigning a single letter grade to indicate risk, the new labeling includes thorough summaries of the risks associated with drug use during pregnancy and lactation, supported by human and animal study data, clinical considerations, and information from pregnancy exposure registries if available. It also adds a new section on females and males of reproductive potential, offering guidance on contraception, fertility, and pregnancy testing when applicable. Additionally, the PLLR requires that labels be updated when new data emerges to ensure the information remains accurate and current, ultimately helping healthcare providers offer more informed counseling to patients [77].

The effects of antibiotics on a developing fetus vary depending on the stage of pregnancy. Before day 31, exposure to harmful substances leads to an all-or-none response, meaning the fetus either survives without defects or does not survive at all. Between days 31 and 71, since this is a crucial period for organ development, the fetus is most vulnerable to birth defects caused by teratogenic drugs. After day 71, while drug exposure can still affect fetal growth and organ function, the risk of major malformations is lower compared to the earlier stage [78,79]. At the moment, in the absence of Category A pharmacological data, clinical practice favors the use of Category B drugs during pregnancy. Antibiotics that are considered safe and can be given during all trimesters of pregnancy, include, among others, β-lactams and their inhibitors, vancomycin, daptomycin, clindamycin, etc. [80]. On the other hand, aminoglycosides and tetracyclines should be reserved for critical cases due to their toxicity to the fetus during all stages of pregnancy [77].

Of note, profound physiological and anatomical changes in nearly every organ system during pregnancy significantly affect the pharmacokinetic and pharmacodynamic properties of medications used by pregnant individuals [81]. Specifically, increased progesterone levels lead to delayed gastric emptying, which often alters the bioavailability of certain drugs. In addition, nausea and vomiting during early stages of pregnancy may impact drug absorption and can result in lower plasma concentrations. Renal blood flow and GFR increase by 50% in the first trimester (by 14 weeks). This may significantly increase the elimination rate of renally cleared medications leading to shorter half-lives. In the same way, this change in pharmacokinetics (PK) can affect the target attainment of antibiotics. Regarding the PK of penicillins in pregnant women, during the second and third trimesters, a higher drug clearance (CL) and larger volume of distribution (Vd) were reported compared to non-pregnant women and pregnant women in the first trimester [82]. Additionally, reduced target attainment was described in pregnant women during the second and third trimesters. The larger Vd and higher CL in second and third trimester pregnant women might warrant higher dosages or a shortening of the dosing interval for penicillin to improve target attainment [82]. However, studies frequently failed to provide dosing advice, even when PK data were available [82].

#### 9.1.2. Empirical Therapy

There are currently no specific guidelines guiding the management of pregnancy-associated IE; therefore, treatment is based on the assumption that the causal organisms are similar to those found in non-pregnant individuals. Empirical antibiotic treatment for IE should be initiated promptly after obtaining three sets of blood cultures at 30-min intervals to ensure accurate pathogen identification [48]. The choice of antibiotics depends on factors such as previous antibiotic use (with consideration of the potential for endemic resistance), valve type (native or prosthetic), timing of prosthetic valve surgery, and whether the infection is community-acquired, nosocomial, or healthcare-associated [48].

Empiric treatment for native valve endocarditis (NVE) and late prosthetic valve (PVE) should provide coverage for staphylococci, streptococci, and enterococci. In patients who have already been on antibiotic therapy, alternative agents should be selected for empiric coverage. Coagulase-negative staphylococci (CoNS) should be included in empiric therapy for PVE but not for NVE. For early PVE or healthcare-associated IE, empiric regimens should target methicillin-resistant staphylococci, enterococci, and preferably non-HACEK Gram-negative organisms. Hence, a suitable empiric regimen for suspected NVE includes vancomycin in combination with ceftriaxone. In cases where there is a concern for oral or gastrointestinal sources of infection, ampicillin-sulbactam may be used as an alternative to ceftriaxone [83]. For patients at risk of nosocomial infections involving *Pseudomonas* species, cefepime or piperacillin–tazobactam may be substituted for ceftriaxone. A carbapenem may be also selected, though carbapenems should be reserved when possible to reduce resistance pressure.

However, use of intravenous vancomycin represents a challenge in pregnant individuals, as vancomycin is generally avoided [48]. A more compatible empirical treatment in NVE or PVE during pregnancy would include daptomycin (instead of vancomycin), especially at doses of 8–10 mg/kg/day when *S. aureus* is suspected or 10–12 mg/kg/day if targeting *Enterococcus* spp., in combination with a second agent, such as ceftriaxone or cefazolin. Ceftriaxone provides strong coverage for streptococci and HACEK organisms, while cefazolin is preferred for methicillin-sensitive *S. aureus* (MSSA).

Once culture and susceptibility data are available, antibiotic therapy should be adjusted accordingly. However, we should bear in mind that antibiotic stewardship at this setting should be carefully balanced against potential fetal harms of de-escalating regimens.

#### 9.1.3. *S. viridans* and *Streptococcus gallolyticus* Group

For patients with IE due to penicillin-susceptible (MIC ≤ 0.12 mcg/mL) oral *Streptococci* or the *Streptococcus gallolyticus* group, the recommended antibiotic regimen includes penicillin G, amoxicillin, or ceftriaxone for 4 weeks NVE and 6 weeks in PVE. For penicillin-allergic patients, desensitization is preferred; if not possible, cephalosporins (for non-anaphylactic reactions) can be used as an alternative regimen [48].

In cases caused by penicillin-resistant *S. viridans* and *S. gallolyticus*, the key point is that all proposed antibiotic regimens include either aminoglycosides, gentamycin, or vancomycin, which are not indicated in pregnancy. Moreover, there is lack of current guidelines supporting daptomycin as an alternative regimen to these resistant cases [48,77]. Data from current literature, which are not specified for pregnancy, support that daptomycin alone is not bactericidal against *Streptococcus mitis* group IE, rapidly inducing high-level resistance, but its efficacy and resistance prevention improves when combined with ceftriaxone or ceftaroline [84,85].

#### 9.1.4. *S. aureus* and Coagulase-Negative Staphylococci

For MSSA NVE, cloxacillin, flucloxacillin, cefotaxime, or cefazolin is recommended for 4–6 weeks. If allergic or intolerant to penicillin, daptomycin combined with ceftaroline or fosfomycin may be considered [48]. The latter combination is optimal for pregnant individuals with MSSA PVE [48].

For methicillin-resistant *S. aureus* (MRSA) NVE, daptomycin alone or in combination with cloxacillin, ceftaroline, or fosfomycin may be considered [12,48]. For MRSA PVE, guidelines recommend a triple regimen treatment, including daptomycin plus rifampin for at least 6 weeks, with gentamicin for the first 2 weeks. Due to the fact that the recommended regimens bear a risk for pregnant individuals, it is important to weigh the cost between maternal health and fetal complications. Other potential safe alternatives in this case include fosfomycin plus imipenem [86], ceftaroline [87], or quinupristin–dalfopristin with or without beta-lactams [88]. However, these data come from small reports and do not exclusively include PVE MRSA. For CoNS PVE, which is often related to a high rate of methicillin resistance and significant valvular complications [89], the recommended regimen does not differ from that of *S. aureus*; however, in the absence of antibiogram, methicillin resistance should be assumed and treated accordingly with daptomycin [48].

#### 9.1.5. *Enterococci* spp.

Effective treatment for enterococcal IE requires prolonged therapy (up to 6 weeks) with synergistic antibiotic combinations. Considering limitations during pregnancy, for non-high-level aminoglycoside-resistant (HLAR) *E. faecalis* IE, the preferred regimen is ceftriaxone plus ampicillin or amoxicillin for 6 weeks. *E. faecium* is often more resistant, with beta-lactam or vancomycin resistance being common, requiring tailored therapy based on susceptibility [90]. Multidrug-resistant enterococci infections may be treated with daptomycin combined with beta-lactams (ampicillin, ertapenem, or ceftaroline) or fosfomycin to prevent daptomycin resistance, but also align with pregnancy safety considerations [48,91].

#### 9.1.6. HACEK and Non-HACEK Gram-Negative Bacteria

Although combinations with alternative regimes are available for HACEK group bacteria, considering limitations during pregnancy, the preferred treatment is ceftriaxone for 4 weeks in NVE and 6 weeks in PVE. If the bacteria do not produce beta-lactamase, ampicillin monotherapy is sufficient according to AHA latest guidelines [12]. However, ESC does state the need for combination therapy with gentamicin in such cases [48]; of note, the latter is considered unsafe in pregnant patients.

Non-HACEK Gram-negative IE, though rare [92], is severe and requires early surgery plus 6 weeks of combination therapy with beta-lactams and aminoglycosides, sometimes supplemented with quinolones or cotrimoxazole. Due to their complexity, bactericidal tests and specialist consultation are recommended, with careful assessment of antimicrobial sensitivities [48].

#### 9.1.7. Fungi

Fungal IE is a rare but serious condition, typically seen in immunocompromised patients, IVDU, or those with prosthetic valves. It is associated with a very high mortality rate, often exceeding 50%, and requires aggressive treatment involving both antifungal therapy and surgical intervention.

IE due to *Candida* spp. is usually treated with liposomal Amphotericin B (or other lipid formulations), with or without the addition of flucytosine or high-dose echinocandin as monotherapy. Long-term suppressive therapy is recommended using oral azoles, particularly fluconazole, and this is sometimes required lifelong to prevent relapse. Similarly, for *Aspergillus* IE, voriconazole is the drug of choice. Liposomal Amphotericin B is reserved for patients at risk for drug interactions with azoles, isolates suspected to be triazole-resistant, or significant hepatotoxicity. At the moment, in pregnancy, only liposomal Amphotericin B is considered relatively safe to use. In any case due to the complexity and severity of fungal endocarditis, management should always involve consultation with a multidisciplinary endocarditis team and include early surgery [48].

Table 1 summarizes the available antibiotic regimens for IE during pregnancy, tailored to the causative pathogen (only regimens considered safe for use during pregnancy included).

#### 9.1.8. Follow-Up and Duration of Therapy

Most individuals with NVE become afebrile within 3–5 days following initiation of effective antibiotic therapy. However, those with *S. aureus* NVE may take longer—typically 5–7 days—to show a reduction in fever. Patients with right-sided IE and septic pulmonary emboli may experience persistent fever for an even longer period. To evaluate the initial microbiologic response, repeat blood cultures should be obtained 48 h after the initiation of antibiotics. It is advisable to continue collecting at least two sets of blood cultures every 24 to 48 h until bacteremia is resolved.

Thorough and repeated physical examinations to monitor for signs of heart failure, embolic events, or other complications is strongly recommended. If new issues arise during treatment—such as emboli, heart failure, conduction abnormalities, or other concerns—a follow-up echocardiogram should be performed to check for progressive valve damage, abscess formation, or fistula development.

The typical antibiotic therapy lasts 4 to 6 weeks for native valve infections, with longer courses for prosthetic valve infections [9] due to the formation of biofilms [48]. Treatment approaches vary depending on the causative pathogen and the nature of the affected valve (native or prosthetic). In non-pregnant individuals, a 2-week intravenous antibiotic therapy could be followed by an oral continuation upon hospital discharge [48]. Outpatient parenteral antibiotic therapy (OPAT) or step-down oral antibiotic therapy has become a viable option in the management of IE once critical infection-related complications are controlled and the patient is clinically stable [48]. However, successful outpatient therapy requires careful patient selection, education, regular clinical monitoring, and strong adherence to the prescribed regimen [48]. Inclusion in an OPAT program requires that patients meet strict clinical, laboratory, and echocardiographic criteria—including clinical stability, absence of endocarditis-related complications, reliable IV access, and sufficient cognitive and social support—to ensure safe and effective outpatient management [93]. Although for the pregnant patient, this step-down approach could show positive outcomes, including reduced hospitalization times, improved patient comfort, and continued clinical effectiveness [48], to the knowledge of these authors, there is paucity of data in pregnancy, hence not currently recommended. Similarly, for patients unable to undergo surgery at specific point during pregnancy, data on long-term suppressive therapy are absent, and in any case, benefits should be carefully weighed against fetal harm from long-term antibiotic administration.

### 9.2. Anticoagulant Therapy

IE during pregnancy is associated with both bleeding and thrombotic events, the latter being frequent among complications [61]. In fact, thromboembolic complications and heart failure seem to be the main cause for maternal morbidity and mortality in some studies [5]. Thromboembolic events are mentioned in case reports [94], some referring to a percentage of 16% in symptomatic embolisms [38]. On the other hand, apart from septic emboli, which can be observed in high percentages (up to 40% of cases) in reported studies, hemorrhagic stroke has also been observed in up to 10% of cases [95].

When selecting an anticoagulation strategy, particularly for individuals with prosthetic heart valves during pregnancy, it is important to carefully balance between maternal and fetal safety. Although warfarin is considered the most effective regimen, it is often related to adverse fetal outcomes [96]. On the other hand, therapeutic dose low molecular weight heparin (LMWH) improves fetal outcomes compared to warfarin but may lead to an increased risk of thromboembolic complications [96]. Reported studies indicate that high doses of warfarin (above 5 mg/day) during the first semester are associated with higher fetal risks, including spontaneous abortion and fetal loss add ref. Meanwhile, the use of heparin results in increased maternal complications such as heart failure, arrhythmias, and IE [97]. As a result, more intensive anticoagulation with higher anti-Xa levels might help reduce the risk of valve thrombosis in pregnant women with mechanical heart valves [96].

### 9.3. Surgical Management

According to the latest ESC guidelines, the three main indications for surgery IE for non-pregnant individuals are heart failure, uncontrolled infection, and the prevention of systemic embolization, particularly to the central nervous system. Heart failure remains the most frequent and urgent reason for surgical intervention, especially in patients with severe valve dysfunction and hemodynamic instability. Uncontrolled infection, in terms of persistent bacteremia, septic shock, or perivalvular complications such as abscesses or pseudoaneurysms, is the second major indication. Finally, surgery is considered to prevent embolic events in patients with large, mobile vegetations, especially during the early phase of antibiotic therapy when embolic risk is highest [48]. However, for pregnant patients, the guidelines propose heart failure due to acute regurgitation or cardiogenic shock as an absolute indication for valve surgery, while uncontrolled infection and prevention of embolism are considered relative indications, requiring an individualized assessment [76].

**Figure 4:**
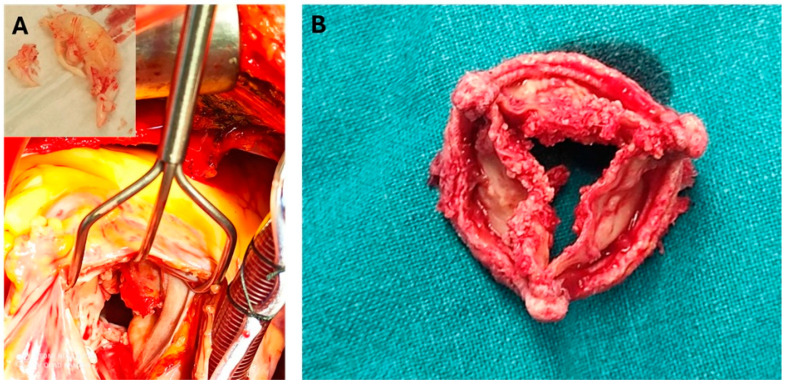
Intraoperative specimens of (**A**) mitral valve endocarditis and (**B**) degenerated bioprosthetic aortic valve following endocarditis.

The decision to proceed with valve surgery during pregnancy should be approached with caution due to significant risks for both the mother and the fetus. A meta-analysis reported maternal complications in 8.8 per 100 pregnancies, neonatal complications in 10.8 per 100, and a pregnancy loss rate of 33.1 per 100, with maternal mortality estimated at 11.2 per 100 [98]. Similarly, a retrospective cohort study highlighted the high risks associated with cardiac surgery during pregnancy, noticing that preoperative factors such as left ventricular ejection fraction (LVEF), pulmonary hypertension (PH), and intraoperative blood loss are reliable predictors of postoperative cardiovascular complications and mortality [99]. The timing of cardiac surgery during pregnancy does not significantly impact maternal mortality; however, fetal mortality is lowest when surgery is performed in the third trimester [100]. Importantly, performing a cesarean section (CS) before cardiac surgery significantly reduces fetal mortality [100]. It is recommended that the management of pregnant individuals with IE should be performed in tertiary centers with a multidisciplinary team [38,76]. In general, surgery should be delayed until infection is resolved. However, in high-risk situations such as cardiogenic shock or refractory HF, which require urgent surgery, a short course of antibiotic treatment should be administered prior to surgery while acknowledging the risk of pulmonary embolism [101]. The ideal window for surgery would be after 28 weeks of pregnancy, as surgery before 24 weeks carries a high risk of fetal death and insufficient growth [102] (Figure 4).

### 9.4. Management of Device-Related Infections

Pacemakers are now frequently encountered in young individuals, including pregnant women, due to the growing use of these devices in younger patients [103]. As a result, device-related infections pose significant diagnostic challenges and may lead to considerable morbidity and mortality. Such infections not only worsen clinical prognosis but also negatively affect patients’ quality of life [104].

The AHA guidelines, which apply primarily to non-pregnant individuals, emphasize that due to limited clinical trial data, the optimal choice and duration of antimicrobial therapy for device-related infections remain unclear. However, early removal of infected devices is associated with better outcomes, and reimplantation should be delayed until all signs of local and systemic infection have resolved. In cases of valvular IE, a delay of up to 14 days is recommended. When reimplantation is postponed, the use of wearable defibrillators and temporary–permanent pacemakers are considered appropriate interim strategies [105].

Transvenous lead extraction (TLE) plays a central role in the management of device-related infections. Timely and effective lead extraction is a critical component of achieving favorable clinical results. Advances in extraction tools and techniques have significantly enhanced the safety and success rates of the procedure. Nonetheless, TLE remains associated with serious risks, including vascular injury and mortality. Emerging strategies, such as the use of jugular and femoral access routes, offer promise for further improving procedural outcomes [106].

Despite these findings, data on the management of device-related infections in pregnant individuals remain limited.

## 10. Antimicrobial Prophylaxis

Antibiotic prophylaxis is commonly recommended in high-risk patients for IE; however, its use in pregnant women requires careful consideration [12]. The main controversies include a lack of research specifically focused on pregnant women, as most studies center on non-pregnant individuals, making it difficult to establish clear guidelines. Additionally, the potential risks of antibiotic use—such as allergic reactions, antibiotic resistance, and disruption of the gut microbiome)—must be balanced against the benefits. Finally, there is uncertainty whether prophylaxis is necessary for non-cardiac procedures (e.g., dental and gastrointestinal) in pregnant women, as evidence supporting its use in this context remains limited.

Several organizations have provided guidelines regarding the use of antibiotic prophylaxis in patients at high risk for IE. In pregnant women, these recommendations generally align with those for non-pregnant individuals, with modifications to account for pregnancy-related physiological changes. The AHA guidelines recommend prophylactic antibiotics for high-risk individuals who are undergoing specific invasive procedures that may introduce bacteria into the bloodstream [12]. In contrast, the National Institute for Health and Care Excellence (NICE) guidelines in the UK advises against the routine use of antibiotic prophylaxis, even in dental procedures [107]. Meanwhile, the ESC guidelines support prophylaxis for high-risk patients undergoing specific procedures such as dental, gastrointestinal, or genitourinary interventions. However, the ESC highlights the need for individualized recommendations in pregnancy, considering factors such as the stage of pregnancy and maternal health status [48,108].

If prophylaxis is selected, administering prophylactic systemic antibiotics within one hour before an incision is the standard of care [48]. The choice of antibiotic for prophylaxis in pregnant women must be made with consideration of both efficacy and safety for the mother and fetus. Common antibiotics used in the prophylaxis of IE are shown in Table 2 (only regimens considered safe for use during pregnancy included).

In any case, the use of antibiotic prophylaxis in high-risk pregnant women must be personalized, making sure the benefits of treatment outweigh any potential risks in the life of the mother and the fetus. The data suggest it is not routine for operations with no entry in the bowel, the vagina, or cervical tissue excision surgery. The available studies show a significantly reduced risk of surgical site infections but do not specify IE. Nevertheless, it is commonly accepted that high-risk IE pregnant women should be given antibiotic prophylaxis [109,110].

## 11. Future Perspectives

The future of IE management in pregnancy will likely focus on enhancing diagnostic accuracy, optimizing treatment strategies and drug delivery, and developing personalized, pregnancy-specific guidelines. First and foremost, development of pregnancy-specific diagnostic criteria that account for the unique physiological changes of gestation is pivotal. Following that, faster and more precise diagnostic tools are crucial for early detection and intervention. Traditional methods like blood cultures and echocardiography remain the gold standard [111], but emerging technologies such as cell-free DNA sequencing (cfDNA) [112,113,114], metagenomic next-generation sequencing (mNGS) [115,116], and polymerase chain reaction (PCR) [117] offer promising alternatives. These methods provide quicker, more sensitive, and non-invasive diagnostics, potentially revolutionizing IE detection in pregnancy [14,112,113,114,115,116,117,118,119]. Additionally, expanding the use of advanced imaging techniques, including contrast-enhanced echocardiography and magnetic resonance imaging adapted for pregnancy, may enhance diagnostic sensitivity while minimizing fetal risk. However, challenges such as high costs, limited accessibility, and the need for standardization must be addressed before routine clinical implementation [14,112,113,114,115,116,117,118,119].

While antibiotics remain the cornerstone of IE treatment, selecting the safest and most effective regimen for pregnant women is a challenge. Even among the limited number of antibiotics considered safe for use in pregnancy, achieving and predicting therapeutic blood concentrations continues to be a subject of ongoing debate. Therapeutic ranges for most medications have been established in non-pregnant populations and remain unvalidated in pregnant women. Extrapolation without accounting for gestational pharmacokinetic–pharmacodynamic changes may lead to suboptimal treatment. Laboratory assays for many drugs are limited, and clinical management should prioritize patient-centered assessment over isolated laboratory values. Despite limited PK/PD studies, there is insufficient evidence from large clinical trials to confirm that therapeutic drug monitoring (TDM) improves maternal or fetal outcomes, and its cost-effectiveness in pregnancy remains unproven [120,121].

Future research is likely to focus on tailored antibiotic therapy, optimizing regimens that are both effective against IE and safe for fetal development. However, even if a molecule were made very large or tightly protein-bound (to reduce placenta transfer), that would likely also reduce its ability to reach and penetrate maternal infection sites effectively. Nonetheless, experimental research currently assesses nanocarrier systems (attaching antibiotics to large nanoparticles that do not cross the placenta easily), targeted drug delivery (releasing antibiotics only at the site of infection, minimizing systemic exposure), and placental efflux transporters (drugs that get pumped back into maternal circulation by placental transport proteins like P-gp) to minimize fetal exposure [122,123]. Emerging approaches, such as sonobactericide therapy—which uses ultrasound-activated lipid-coated microbubbles to degrade biofilms—show promise for non-invasive bacterial eradication [124]. Additionally, novel antimicrobial agents, including non-antibiotic direct lytic therapies, could provide alternative treatment options, particularly in cases resistant to traditional antibiotics [125].

The complexity of IE in pregnancy necessitates a collaborative approach involving cardiologists, infectious disease specialists, obstetricians, neonatologists, and microbiologists. Establishing dedicated endocarditis teams will help improve diagnosis, management, and outcomes for pregnant women with IE [48,126,127,128,129,130,131].

## 12. Conclusions

IE during pregnancy is rare but poses significant risks to both maternal and fetal health, with high mortality rates. Pregnancy-related physiological changes, such as increased cardiac output and immune modulation, may increase susceptibility to IE. While diagnostic procedures used in pregnant women are similar to those in non-pregnant individuals, treatment options are limited due to the potential fetal toxicity of certain antibiotics. Even though traditional methods like blood cultures and echocardiography remain the gold standard, newer molecular techniques offer faster, more sensitive, and less invasive alternatives for early detection. Managing IE in pregnancy requires a multidisciplinary approach, involving cardiologists, infectious disease specialists, obstetricians, neonatologists, and cardiovascular surgeons.

Future research should focus in increasing our knowledge of pathophysiologic alterations in pregnancy, but also our understanding of long-term maternal and fetal outcomes. Launching pharmacokinetic and pharmacodynamic studies so as to refine antibiotic selection and dosing, but also longitudinal cohort studies to track developmental and health outcomes in children exposed to maternal IE and its treatments would provide invaluable data. Establishing national and international registries dedicated to pregnancy-associated IE is critical, in order to collect real-world evidence that will inform future guidelines.

## Figures and Tables

**Table 1:** Antibiotic regimens used for IE during pregnancy based on causative pathogen.

Pathogen	Recommended Regimen	Consideration During Pregnancy
Empiric Treatment	NVE: Daptomycin (8–10 mg/kg/day IV) or (10–12 mg/kg/day) + Ceftriaxone (2 g/day IV) OR Cefazolin (6 g/day IV in 3 doses) PVE: Same regimens apply for NVE and PVE	
*S. viridans* and *S. gallolyticus*	NVE: Penicillin G (12–18 million U/day IV in 4–6 doses or continuous) OR Amoxicillin (12 g/day IV in 4–6 doses) OR Ceftriaxone (2 g/day IV in 1 dose) for 4 weeks PVE: Same regimens for 6 weeks	
Penicillin-resistant *S. viridans* and *Streptococcus gallolyticus*	NVE: Penicillin G (24 million U/day IV) OR Amoxicillin (12 g/day IV) OR Ceftriaxone (2 g/day IV) + *Gentamicin (3 mg/kg/day IV) for 2 weeks PVE: Same regimens as NVE but for 6 weeks	*Gentamicin and vancomycin generally avoided in pregnancy. Consider ceftriaxone + daptomycin OR ceftaroline, but efficacy data in pregnancy are limited.
MSSA	NVE: Flucloxacillin (12 g/day IV) OR Cefazolin (6 g/day IV) for 4–6 weeks	
	PVE: Daptomycin (10 mg/kg/day IV) + Ceftaroline (1800 mg/day IV in 3 doses) OR Fosfomycin (8–12 g/day IV)	
MRSA and CoNS	NVE: Daptomycin (10 mg/kg/day IV) + Cloxacillin (12 g/day IV) OR Ceftaroline (1800 mg/day IV in 3 doses) OR Fosfomycin (8–12 g/day IV)	
	PVE: Daptomycin (10 mg/kg/day IV) + *Rifampicin + *Gentamicin (3 mg/kg/day IV) for 6 weeks	*Rifampicin and gentamicin carry fetal risks. Weigh maternal benefit versus fetal risk. Other potential safe alternatives include fosfomycin plus imipenem, ceftaroline, or quinupristin–dalfopristin with or without beta-lactams, although these data from reports do not exclusively include PVE MRSA.
*Enterococcus* spp.		
non-HLAR	Ampicillin (200 mg/kg/day) or Amoxicillin (12 g/day IV in 4–6 doses) + Ceftriaxone (4 g/day IV in 2 doses) for 6 weeks	
HLAR	Same regimen as non-HLAR Multiresistant: Daptomycin (10 mg/kg/day IV) + one of Ampicillin (200 mg/kg/day), Ertapenem (2 g/day IV), Ceftaroline (1800 mg/day IV in 3 doses), or Fosfomycin (8–12 g/day IV)	
HACEK Group	Ceftriaxone (2 g/day IV for 4 weeks—NVE, 6 weeks—PVE) If beta-lactamase-negative: Ampicillin monotherapy possible	
Non-HACEK Gram-negative	Beta-lactams + *Aminoglycosides for 6 weeks; sometimes + *Quinolones or *Cotrimoxazole	Many agents carry risk: aminoglycosides, quinolones, or cotrimoxazole. Requires early surgery and specialist consultation
Fungi (e.g., Candida, Aspergillus)	Liposomal Amphotericin B (3–5 mg/kg IV)	

IE: Infective endocarditis; NVE: Native valve endocarditis; PVE: Prosthetic valve endocarditis; MSSA: Methicillin-sensitive *Staphylococcus aureus*; MRSA: Methicillin-resistant *Staphylococcus aureus*; CoNS: Coagulase-negative staphylococci; HLAR: High-level aminoglycoside resistance; IV: Intravenous; mg: Milligrams; g: Grams; U: Units; HACEK: *Haemophilus* spp., *Aggregatibacter* spp., *Cardiobacterium hominis*, *Eikenella corrodens*, *Kingella* spp. * Indicates that the treatment or intervention is not approved for use in pregnant individuals; however, it may be used when it is considered the only viable or necessary option based on clinical judgment

**Table 2:** Recommended regimens for IE prevention for high-risk pregnant patients (note: there are no specific recommendations for pregnant women).

Antibiotic	Dose	Route of Administration	Time Before Incision
Amoxicillin	2 g	Orally	30–60 min
Ampicillin	2 g	i.m. or i.v.	30–60 min
Cefazolin or Ceftriaxone	1 g	i.m. or i.v.	30–60 min
Cephaxelin	2 g	Orally	30–60 min
Azithromycin or Clarithromycin	500 mg	Orally	30–60 min

IE: Infective endocarditis; i.m.: Intramuscular; i.v.: Intravenous; min: Minutes.

## Supplementary Materials

The following supporting information can be downloaded at: https://www.mdpi.com/article/10.3390/jcm14124262/s1, Table S1: Studies of pregnancy-associated IE and respective outcomes [6,7,13,39,72,73,74,75,76,77].

## Abbreviations

The following abbreviations are used in this manuscript:

IE	Infective Endocarditis
*S. aureus*	*Staphylococcus aureus*
IUGR	Intrauterine Growth Restriction
IVDU	Intravenous Drug Use
EVs	Extracellular Vesicles
HLA-G	Human Leukocyte Antigen G
NK	Natural Killer (cells)
miRNAs	MicroRNAs
Treg	Regulatory T cell
Th1	T helper type 1
Th2	T helper type 2
IL-10	Interleukin 10
*S. viridans*	*Streptococcus viridans*
*E. faecalis*	*Enterococcus faecalis*
HACEK	*Haemophilus* spp., *Aggregatibacter actinomycetemcomitans*, *Cardiobacterium hominis*, *Eikenella corrodens*, *Kingella kingae*
ESC	European Society of Cardiology
AHA	American Heart Association
ACOG	American College of Obstetricians and Gynecologists
TTE	Transthoracic Echocardiography
TEE	Transesophageal Echocardiography
MRI	Magnetic Resonance Imaging
CT	Computed Tomography
FDA	Food and Drug Administration
PK	Pharmacokinetics
CL	Clearance
Vd	Volume of Distribution
NVE	Native Valve Endocarditis
PVE	Prosthetic Valve Endocarditis
CoNS	Coagulase-Negative Staphylococci
HLAR	High-Level Aminoglycoside Resistance
MSSA	Methicillin-Sensitive *Staphylococcus aureus*
MIC	Minimum Inhibitory Concentration
NICE	National Institute for Health and Care Excellence
OPAT	Outpatient Parenteral Antibiotic Therapy
CS	Cesarean Section
*E. faecium*	*Enterococcus faecium*
cfDNA	Cell-Free DNA
mNGS	Metagenomic Next-Generation Sequencing
PCR	Polymerase Chain Reaction
LMWH	Low Molecular Weight Heparin
i.v.	Intravenous
i.m.	Intramuscular
min	Minutes
LVEF	Left Ventricular Ejection Fraction
PH	Pulmonary Hypertension
PLLR	Pregnancy and Lactation Labeling Rule
TLE	Transvenous Lead Extraction
FIRS	Fetal Inflammatory Response Syndrome
