# Supplementary material for: Infective Endocarditis During Pregnancy: Challenges and Future Directions
Source: J Clin Med. 2025 Jun 16;14(12):4262. doi: 10.3390/jcm14124262 (PMC12194181; doi:10.3390/jcm14124262)
Supplement: Supplementary file 1 [file jcm-14-04262-s001.zip › jcm-3651797-supplementary.pdf]

Journal of Clinical Medicine

MDPI

Supp Table S1: Studies of pregnancy-associated IE and respective outcomes.

**Reference**	Time and place conducted	Type of study and Number of cases and study groups	Aim-Compared groups	Pregnancy outcomes	Neonatal outcome	Maternal outcomes
**Schwartz et al. 2020 [73]**	USA, 1999-2014	Retrospective cohort study, 475 IE cases among 13,219,726 deliveries	Risk factors associated with and without IE and effect on maternal mortality.	NR	NR	• Maternal mortality: 5.3% in IE vs. 0.003% in other pregnancies<br>• Valve replacement in 11.5% of cases<br>• Higher death risk with: Acute renal failure, Myocardial infarction, Sepsis
**Morelli et al. 2020 [77]**	USA, 2013-2019	Single centre retrospective review<br>26 pregnant patients with S.aureus bacteraemia,<br>15 of whom with IE.	Primary outcomes: Maternal and fetal in hospital mortality<br>Secondary outcomes: Rate of 6month maternal readmission.	NR	• 16 (73%) babies required neonatal ICU care<br>• 4(16%) infants/fetuses died during hospitalization	• ICU stay and ventilation more common in IE<br>• Death: 1 patient (4%) during hospitalization<br>• Readmission: 27% within 6 months for infection<br>• Septic emboli in all (15/15)<br>(80% needed ICU for sepsis/shock or intubation)<br>• Valve complications: Tricuspid vegetations >2 cm with severe regurgitation (7%)<br>Mitral vegetations >1 cm with severe regurgitation (7%)<br>One patient with Aortic insufficiency progression

**Adesomo et al. 2020 [13]**	USA, 2012-2019	Single centre retrospective study<br>12 pregnancies	Management and clinical implications of IVDU related IE in pregnancy.	Average GA: 37 weeks	• NICU admission for neonatal abstinence syndrome: 4/10 (40%)<br>• Fetal losses: 2 (16%)<br>{One at 19 weeks (post–maternal cardiac surgery)<br>One at 38 weeks (placental abruption)}	• Maternal death: 1 case (8.3%)<br>Occurred after urgent cardiac surgery for heart failure due to:<br>o Severe aortic & tricuspid valve vegetations<br>o Ventricular septal abscess
**Dagher et al. 2021 [72]**	United States 2015-2018	National cohort study<br>Cases: 12,602 reproductive-aged females with IE; 382 (3%) maternity-associated<br>Study Groups: 117 cases during delivery hospitalization (0.00002% of all deliveries)<br>Antepartum IE: 39.1%<br>Postpartum IE: 19.0%	Clinical outcomes of maternity-associated IE vs nonmaternity-associated IE and of obstetric patients who delivered with IE vs without IE.	Preterm birth: 55.7% (avg. GA: 32.4 ± 5.1 weeks)<br><br>Early/abnormal pregnancies: 12.5%<br><br>C-section: ~50%, mostly preterm (avg. GA: 32.4 weeks)<br><br>IE at delivery linked to higher preterm rate (55.7% vs 10.1%)	Concurrent IE was associated with worse fetal outcomes such as stillbirths	• Maternal mortality: 10.6% overall<br>• 60-day mortality highest in delivery subgroup<br>• Valve replacement most common in postpartum group<br>• Main postpartum complications:<br>o Cerebrovascular thrombosis<br>o GI thrombosis<br>o Obstetric clots
**Sinner et al. 2021 [6]**	USA, 2009-2019	Single centre retrospective review<br>19 females with IE during pregnancy	Contemporary outcomes of IE during pregnancy vs historical reports.	Median GA at birth: 36 + 5 weeks	Fetal mortality: 0%<br><br>2/3 of infants: low birth weight and the majority suffered from neonatal abstinence and respiratory distress syndrome	Maternal mortality: 5%

**Shapero et al. 2022 [7]**	Magee Women's Hospital at UPMC, USA, one year study 2020-2021	Single centre, retrospective case series<br>6 pregnant or 30-day post-partum IVDU patients with IE	Management of IE in pregnancy	Preterm delivery rate high (avg. GA: 32.4 weeks for 4 live births)<br><br>1 miscarriage at 10 weeks<br><br>1 lost to follow-up<br><br>Deliveries: 3 vaginal,<br>1 emergent C-section	Fetal mortality: 0%<br><br>Low birth weight: 2 out of 3 infants (average 1816 g)<br><br>Neonatal complications:<br>• Neonatal abstinence syndrome (1 infant)<br>• Respiratory distress syndrome: 2 infants<br>• Intraventricular hemorrhage: 2 infants<br>• Low APGAR scores<br>• NICU admission: 3 out of 4 live births	• Maternal mortality: 0%<br><br>• Embolic complications: 100%<br>o 5 septic pulmonary emboli<br>o 3 cerebral emboli<br><br>• ICU & ventilation: 5 patients (83%)
**Escolà-Vergé et al. 2022 [39]**	14 hospitals (11 from France, 1 from Spain, 1 from Argentina and 1 from Brazil), 21-year study 2000-2021	Multinational retrospective study, 25 cases in non-IVDU pregnant women	Maternal mortality and pregnancy-related complications	Pregnancy complications: 16/25 (64%)<br><br>3 losses (<24 weeks)<br><br>9 urgent C-sections (2 emergency)<br><br>11 preterm births	Fetal mortality: 16%<br>1 Fetal death (≥24 weeks)	Maternal death: 1 (4%) during surgery<br>Valvular regurgitation (mod/severe): 16 (64%)<br>Perivalvular abscess: 4 (16%)<br>IE complications: 20 (80%)<br>Heart failure: 13 (52%)<br>Non-stroke embolism: 4 (16%)<br>Acute renal failure: 1 (4%)

**Shapero et al. 2023 [75]**	USA, 2014-2021	single centre retrospective review,<br>180 patients with IE of which 34 were pregnant.	Primary (in-hospital, 1-year, and 2-year mortality and valve replacement/repair) and Secondary (valve surgeries, a thromboembolic event, arrhythmia, intubation, use of mechanical support, use of extra corporeal membrane oxygenation, intensive care unit<br>admission, and length of ICU and hospital stay)	Preterm delivery rate : 50% (mean GA: 35 weeks)	Live births: 76.4%<br>Perinatal mortality was 19.2%<br><br>NICU: 68%	Pregnant vs in non pregnant patients<br>In hospital 1- and 2- year mortality: 2.9% vs 9.6%<br>Rates of valve replacement 32.4% vs 29%<br>2-year mortality: 20.6% vs 17.8%<br>Rates of pulmonary emboli: 17.6% vs 7.5%<br>Arrhythmia: 17.6% vs 9.6%
**Countouris et al. 2024 [76]**	USA 2015-2018.	Single centre case series study, 9 patients	To assess risk factors and outcomes of peripartum infective endocarditis, primarily in patients with intravenous drug use (89%)	Preterm delivery: 44% (average GA 35 weeks)<br>89% delivered vaginally<br>one required an emergent c-section	No fetal mortality (0%).<br>NICU: 3 newborns (43%)	Maternal mortality: 2 cases (22%)<br><br>Valve involvement: Tricuspid (56%), Mitral + Tricuspid (22%)<br><br>Major complications:<br>Shock: 33%<br>Mechanical ventilation: 44%<br>Septic emboli: 67%<br>Noncardiac abscesses: 33%
**Adams et al. 2024 [74]**	5 tertiary-care hospitals in London, Ontario, and Regina, Saskatchewan, Canada, 2007-2018	Retrospective cohort study,<br>430 IVDU patients with IE o (220 were women and 11 were pregnant)	Identification of clinical characteristics and outcomes of IE among IVDU women	46% were terminated (60% electively)<br>Miscarriages 18%<br>Vaginal births 83%<br>Caesarean births 17%	6 live births (mean GA: 35.3 weeks)<br>Fetus Mortality: 25%<br>NICU: 33%	Maternal Mortality: 27% (Lower Mortality with right-sided heart disease, higher with congestive heart failure)

IE : Infective Endocarditis , IVDU : Intravenous Drug Use, NICU : Neonatal Intesive Care Unit , ICU : Intensive Care Unit, USA: United States of America NR: Not Reported, C-section: cesarean section, GA: Gestational Age
